# Bioinspired polypyrrole based fibrillary artificial muscle with actuation and intrinsic sensing capabilities

**DOI:** 10.1038/s41598-022-18955-6

**Published:** 2022-09-02

**Authors:** Mihaela Beregoi, Samuel Beaumont, Alexandru Evanghelidis, Toribio F. Otero, Ionut Enculescu

**Affiliations:** 1 Multifunctional Materials and Structures Laboratory, National Institute of Materials Physics, Atomistilor Str. 405A, 077125 Magurele, Romania; 2 Laboratory of Electrochemistry Intelligent Materials and Devices, Technical University of Cartagena, Campus Alfonso XIII, 30203 Cartagena, Spain; 3 Present Address: Department of Chemistry and Materials, Faculty of Textile Science and Technology, Shinshu University, Ueda Campus, 3-15-1 Tokida, Ueda, Japan

**Keywords:** Actuators, Sensors and biosensors

## Abstract

A non-conventional, bioinspired device based on polypyrrole coated electrospun fibrous microstructures, which simultaneously works as artificial muscle and mechanical sensor is reported. Fibrous morphology is preferred due to its high active surface which can improve the actuation/sensing properties, its preparation still being challenging. Thus, a simple fabrication algorithm based on electrospinning, sputtering deposition and electrochemical polymerization produced electroactive aligned ribbon meshes with analogous characteristics as natural muscle fibers. These can simultaneously generate a movement (by applying an electric current/potential) and sense the effort of holding weights (by measuring the potential/current while holding objects up to 21.1 mg). Electroactivity was consisting in a fast bending/curling motion, depending on the fiber strip width. The amplitude of the movement decreases by increasing the load, a behavior similar with natural muscles. Moreover, when different weights were hung on the device, it senses the load modification, demonstrating a sensitivity of about 7 mV/mg for oxidation and − 4 mV/mg for reduction. These results are important since simultaneous actuation and sensitivity are essential for complex activity. Such devices with multiple functionalities can open new possibilities of applications as e.g. smart prosthesis or lifelike robots.

## Introduction

The development of nature inspired, soft actuating materials using simple, straightforward techniques has progressively increased during the past decade. The motivation is the disruptive potential for applications of devices with complex actuation functions, similar to those of living organisms. A notable example of this is represented by the field of artificial muscle research where devices fabricated using classical architectures^1–3^ are replaced by faster responding, highly flexible and mechanically stable while simultaneously sensitive materials^4,5^ seek to closely mimic the natural muscles functions. Consequently, various stimuli responsive components were integrated in bioinspired devices such as conducting polymers^6,7^, graphene^8^, carbon nanotubes^9^, dielectric elastomers^10^, etc. and some of them have also sensing abilities. In general, the sensing functions were added to the muscle^11–13^ and therefore few reports about artificial muscles with intrinsic sensing properties^4,5,14^ were presented in scientific literature.

It was demonstrated that artificial muscles with sensing abilities can be fabricated by utilizing conducting polymers, especially polypyrrole (PPy), certain similarities to natural muscles being responsible for this function. From biomimetic point of view, almost every mammalian muscle act as integrated sensor/actuator, due to its sensory receptors, being enable to adjudge the heaviness, stiffness, position, force, etc.^15,16^ A comprehensive comparison between how the natural muscles and those based on PPy behave, can be found in Ref.^17^ The electrochemical reactions that generate the movement, more precisely the ionic exchange between the liquid electrolyte and the PPy film emerging from applying a current/potential can be influenced by numerous parameters. It was reported that PPy based bilayer and trilayer actuators can also be used as physical (mechanical^18^ or tactile^19,20^) or chemical (current, concentration, temperature, electrolyte type^21,22^) sensors. Although some progress in developing intrinsically sensing artificial muscles has been made, improvements are still needed for obtaining more reactive, highly sensitive devices suited for applications.

One approach in order to obtain improved performances is to replace the conducting polymer films with fibrous networks for increasing the active surface and flexibility, thus facilitating the ionic exchange and hence generating highly responsive devices. A fabrication way to obtain fibrous materials with some morphological features similar to animal muscles is the electrospinning technique. In a conventional electrospinning setup, a polymer solution is easily converted into micrometer and submicrometer thin solid fibers due to solvent evaporation^23,24^. This technique allows obtaining random or aligned polymer fiber webs with tailored characteristics which, after functionalization could work as contractile microfibers (similar in diameter with natural myofibrils) and can be packed in parallel bundles (similar with skeletal muscle fibers)^25,26^.

The current work deals with the mechanical sensing properties of a fibrous artificial muscle, which mimics the morphology of natural muscle fibers. The fibrillary structures were obtained through electrospinning and coated with a thin metal layer. Further, the electroactive material (PPy) was deposited onto the metallic layer by electropolymerization in such a way that the PPy covers the microribbons while preserving the morphology and therefore the high active area of the electrospun networks. The actuation and sensing abilities were tested by using a liquid electrolyte and several hung masses, which were designed to simulate an increasing opposing force to the movement of the actuation, in a similar way as performed in previous works^19,20^. The material shows the potential to update the old-fashioned actuation only artificial muscles towards new fibrillary self-sensing ones, coming closer to animal muscles.

## Results

### Artificial muscle actuation behavior

The prepared artificial muscle which consisting in a strip of electrospun microribbons covered with thin gold and PPy layers was morphologically characterized and its actuating and sensing properties were analyzed by using electrochemical techniques.

**Figure 1:**
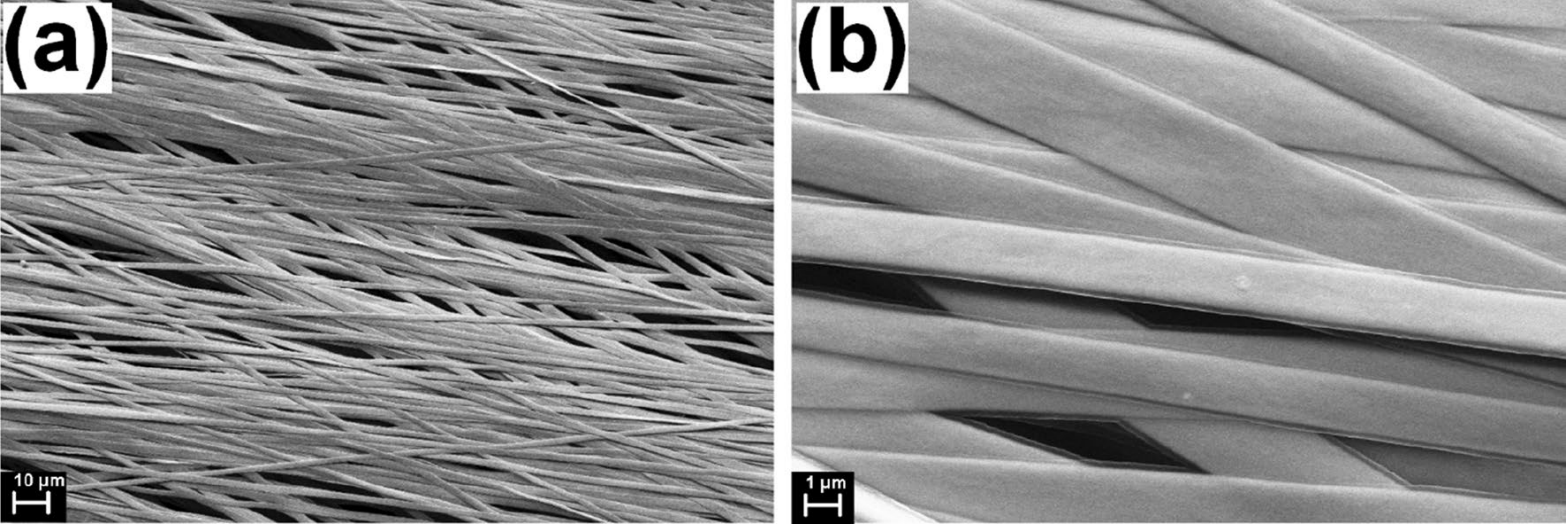
SEM images at two magnifications of Au/PPy coated electrospun microribbons.

The morphological characterization revealed partially aligned flat fibers with width around 1 μm which are uniformly covered with gold and PPy thin layers, as can be seen in Fig. 1. From SEM estimations, the gold layer has a thickness of about 200 nm, while PPy film ⁓ 200 nm.

The actuation properties of the electrochemical artificial muscles depend on various parameters such as electrolyte concentration, its pH or temperature, mesh strip width, applied potential/current range, conditions of applying potential/current. The microribbons covered with a thin layer of gold and PPy are anisotropic structures and since they are coved only on one side with the electroactive material, each microribbon (that have about 1 μm in width) acts as a bilayer actuator and a mesh strip (with sizes of about 0.1 × 2 cm width × length) containing hundreds of such bilayer structures.

**Figure 2:**
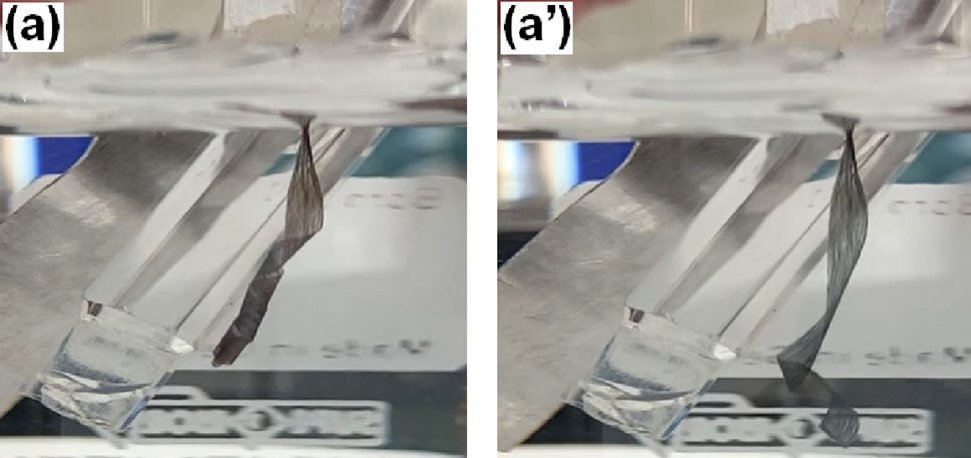
Snapshots taken while applying a square potential to the material without attachment of any mass. The artificial muscle performs a reversible (**a**) clock-wise movement during reduction and a (**a′**) anticlock-wise movement during the oxidation.

As described in a previous work^27^, a curling movement is expected during the application of a constant current or potential. On one hand, when the artificial muscle has not attached any mass, an anticlockwise curling movement was observed during the application of a constant anodic potential and a clock-wise curling movement was observed during the application of a constant cathodic potential. The movement was reversible during the square potential waves, showing a more pronounced curling motion at the reduction phase. Figure 2 presents two snapshots from a video recorded while the square potential wave was applied to the material without any mass attached. Figure 2a depicts the end of the movement after reduction, while Fig. 2a′ shows the end of the movement after oxidation. Video S1 from Supplementary Information shows the full movement during two consecutive periods of the square potential applied. The actuation speed depends on how fast/slow of potential/current is applied, thus in this particular case (Fig. 2 and Video S1), the movement was generated by registering a CV at a scan rate of 100 mV s^−1^ and using 0.1 M LiClO_4_ as electrolyte.

When a mass of 5.3 mg is attached to the microribbon strip, a lower amplitude movement is observed, as shown in Video S2. As the attached mass increases the movement completely disappears.

### Artificial muscle mechanical self-sensing properties

The conventional method for characterizing actuation while mechanical perturbation is applied is using a load cell. However, due to the particular dimensions and mechanical properties (specifically high flexibility and light weight) of the PPy microribbon artificial muscle, creating an experimental setup using a load cell fitted for this task is difficult to achieve. As well, the type of spiraling-grabbing movement generated by the actuation is naturally suited to experimentation using free weights. Such experiments are commonly used in actuator research^28,29^, as they can be useful to illustrate practical functionality in a visually impactful way but, also to gain more complex information about the artificial muscle.

**Figure 3:**
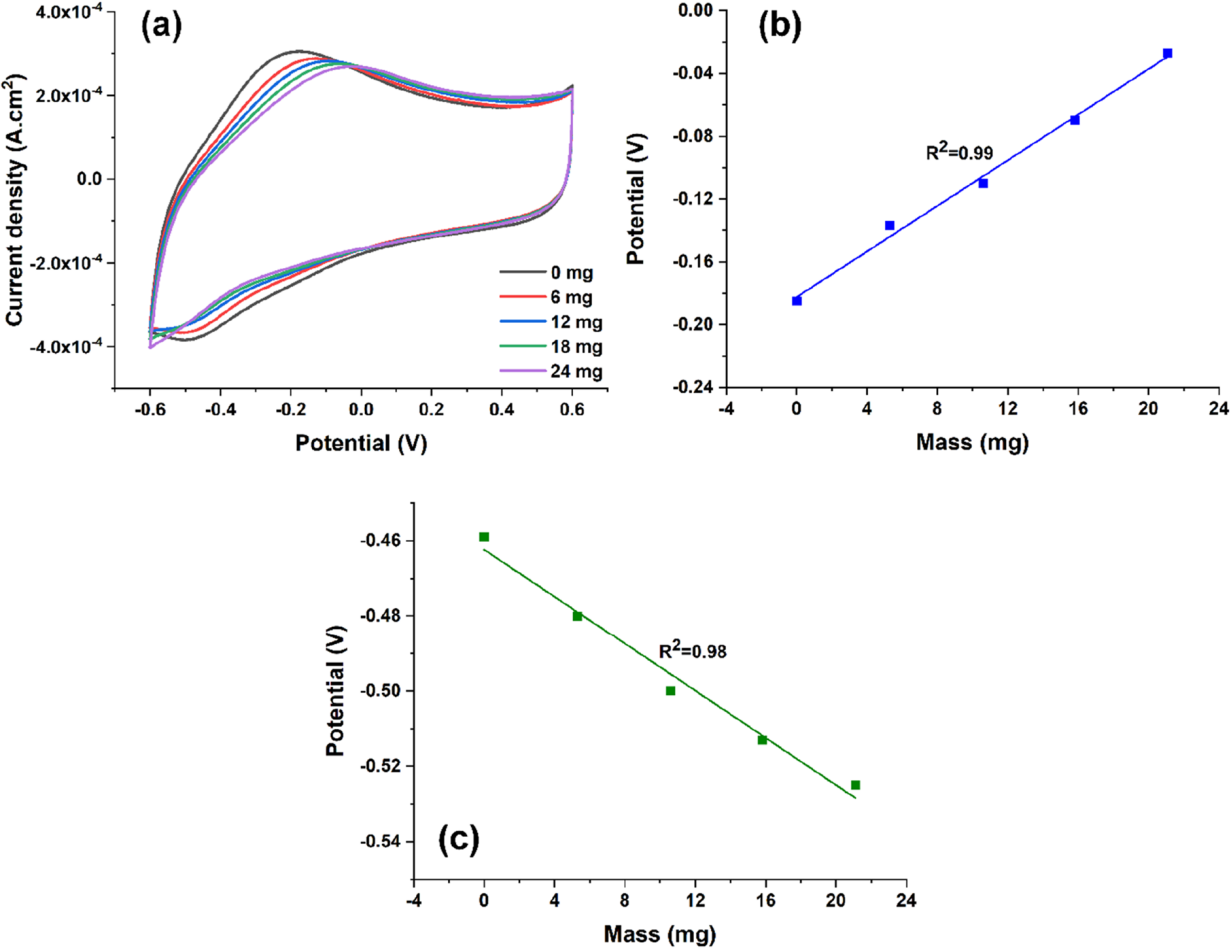
(**a**) Cyclic voltammograms recorded in 0.1 M LiClO_4_ when various masses on the microribbon strip were clung; variation of (**b**) anodic and (**c**) cathodic potentials as function of the attached mass.

In this context, the intrinsic-sensing capabilities of the artificial muscle were monitored during the actuation using cyclic voltammetry, square waves chronopotentiometry and EIS techniques. Thus, CVs in 0.1 M LiClO_4_ aqueous solution were recorded while mechanical stress was applied and are presented in Fig. 3a.

In the CV plots, one pair of anodic/cathodic peaks can be found specific to the oxidation/reduction of PPy film. By hanging different weights on the artificial muscle, the oxidation potentials shift to more electropositive potentials, while potential peaks associated with reduction process shift to more electronegative potentials. As well, the recorded current peak decreases by increasing the clung mass. A representation of the shifted potentials as a function of the attached mass is plotted in Fig. 3b,c.

**Figure 4:**
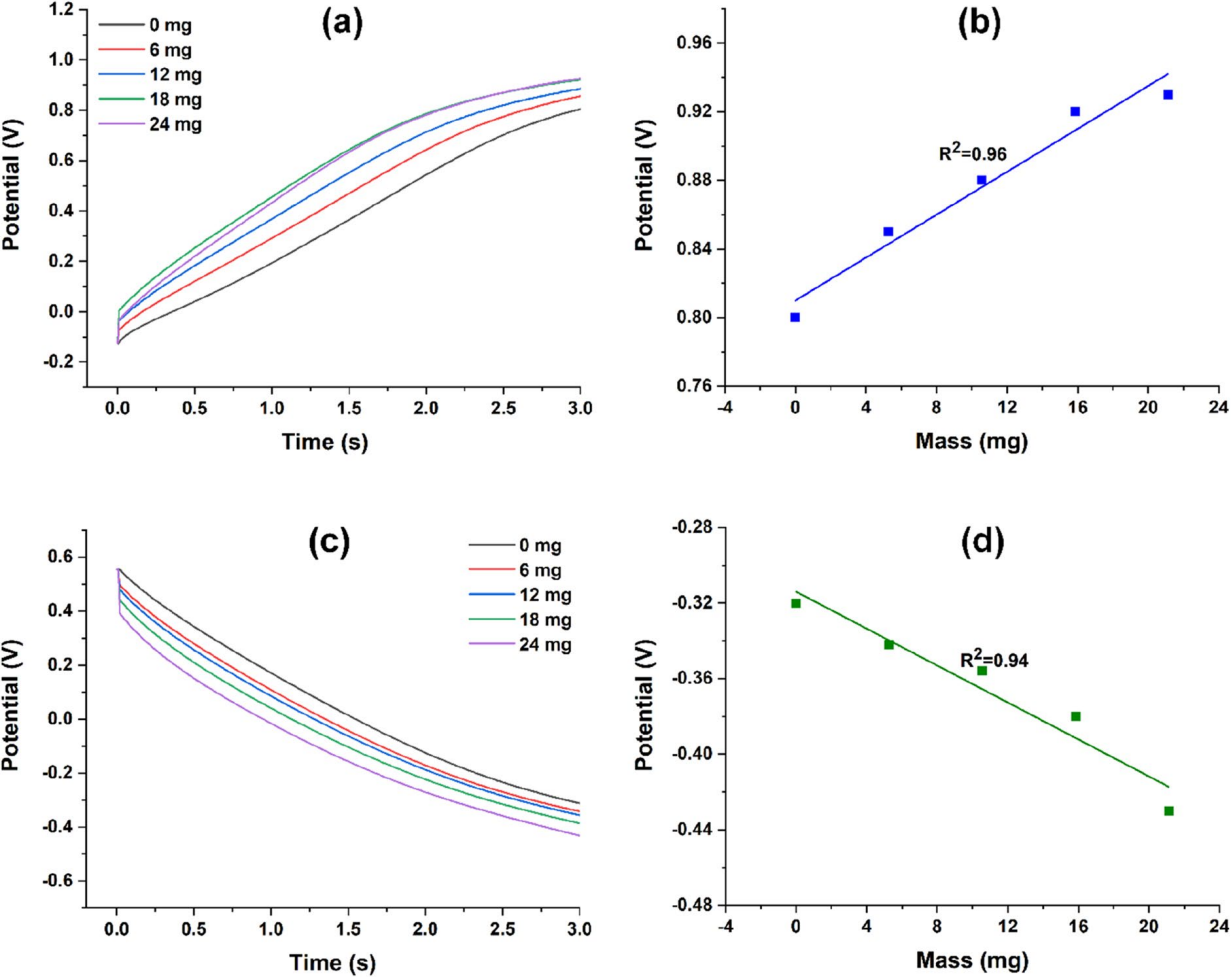
Evolution of the potential reached by the artificial muscle during application of a square current pulse of ± 0.5 mA, obtaining the (**a**,**b**) anodic and (**c**,**d**) cathodic chronopotentiograms for each studied weight.

In Fig. 4a,c, the anodic and cathodic chronopotentiometric responses while current pulses of ± 0.5 mA for 3 s are applied are shown for each mass. For a comparison purposes, a normalization of the data was done taking as a baseline the potential reached by the material for the first applied cathodic current (Fig. 4a) or applied anodic current (Fig. 4c).

An increase (shift towards more electropositive values) in potential is observed for both anodic and cathodic responses as the mass rises. A representation of the potentials recorded versus load during the mechanical stress is presented in Fig. 4b,d.

**Figure 5:**
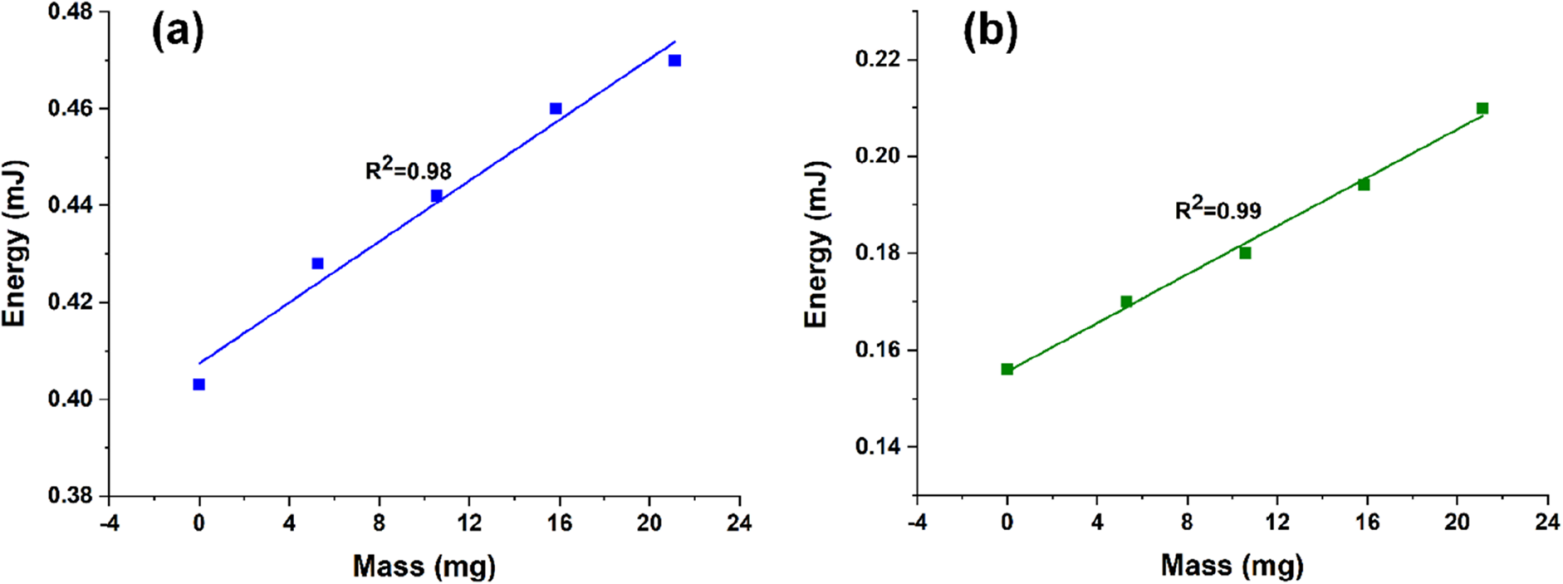
Energy consumed by the electrochemical reactions as function of the attached weight for (**a**) oxidation and (**b**) reduction processes.

The representation of consumed energy hanging different weights on the microribbon strip for the oxidation and reduction processes are displayed in Fig. 5a,b. Similar with recorded potential, the energy consumed by the electrochemical reactions increases by rising the load.

**Figure 6:**
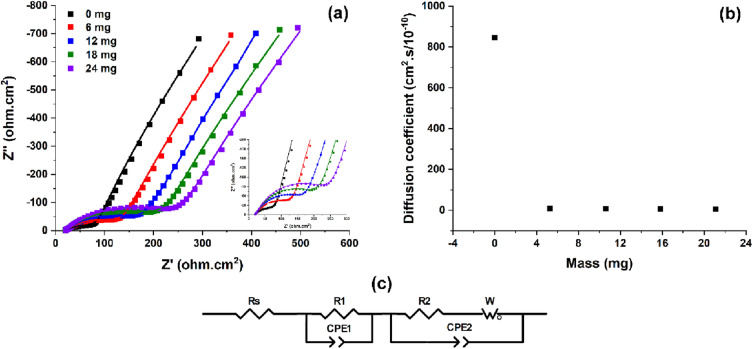
(**a**) Nyquist plots registered by changing the clung weigh and (**b**) the corresponding diffusion coefficients estimated for each mass; (**c**) equivalent electrical circuit used for fitting the EIS results and determining the electrode parameters; Inset: zoom on the impedance semicircles.

A set of EIS measurements were performed in order to quantify how the load influences a set of specific electrode parameters. It is noticeable that since the so-named PPy film is an ensemble of curly polymeric chains, whose actuation is due to the cooperative actuation of every single chain during redox reactions (a deeper explanation is shown in “Discussion” section), there is not a true single double layer because there is not a true single film interface. With this in mind, the recorded EIS experimental data were interpreted by using an approximated electrical equivalent circuit presented in Fig. 6c. The circuit consists of series connected elements (R_s_—charge transfer resistance for the electrolyte resistance, R_1_—charge transfer resistance corresponding to PPy/electrolyte virtual interface, R_2_—charge transfer resistance of Au/PPy virtual interface and W_0_—Warburg semi-finite impedance correlated with the diffusional part characteristic to low frequencies) in parallel with two constant phase elements (CPE, appearing as double layer capacitances of the two mentioned interfaces).

In Fig. 6a (inset representing a zoom on the semicircles) are presented thee Nyquist plots recorded while hanging different weights (up to 21.1 mg) on the artificial muscle. The complex plane plots exhibit one low frequency region assigned to the ions migration described by the Warburg diffusion and one high frequency region related to charge transfer mechanism. By increasing the load, the semicircle radius increases. In Fig. 6b the diffusion coefficients computed by using the time constants emerging from fitting the experimental data for each mass are presented.

A large difference between the diffusion coefficient for unloaded artificial muscle and loaded one can be observed. Further a slight change of the diffusion coefficient values appears when increasing the load.

## Discussion

When the PPy coated microribbon strips are immersed into an electrolyte solution and a square current or potential signal is applied, p-doping and p-dedoping processes take place i.e. reversible oxidation and reduction of polymeric chains^30,31^. The electrochemical reactions of the polymer induce the conformational movement of the chains, the microscopic cooperative actuation leading to the macroscopic swelling and shrinking of the polymer structure during oxidation and reduction by the subsequent lodging and expelling of electrolyte molecules, respectively^32,33^. Since the actuation properties are a consequence of the microscopic movements of swelling and shrinking of every single monomer chain, independently of the structure, the cooperative actuation creates a macroscopic movement, which will depend on the isotropy of the material. The presented models are generally valid for all bilayer actuator types (isotropic or anisotropic), so they can apply also for this material.

In this particular case the fibrous structure of the material i.e. a high aspect ratio architecture leads to a macroscopic curling movement, being anti-clockwise during oxidation and clockwise during reduction (Fig. 2a,b). The high speed (15 s for a complete actuation cycle, see Video S1) and large amplitude of these processes are favored by the high exchange active surface of the fibrillary artificial muscle. For upholding these statements, a comparison between this results and few latest works regarding actuators based on polypyrrole is presented in Table 1.

**Table 1:** Actuation performances of PPy based actuators.

Material	Bending		References
	Angle/displacement	Speed	
Poly(acrylic acid)/polypyrrole hydrogel	Bending angle: 22.02 ± 2.07°	150 s	^34^
Ti_3_C_2_T_x_/PPy bilayer film	Displacement: 30 mm at 0.005 Hz	250 s	^35^
Polyurethane/polypyrrole-p-toluenesulfonate nanofibers	Bending angle: 141°	–	^36^
Silk-PPy film	Bending angle: 30°	120 s	^37^
Silk-ppy polymer composite trilayer actuators	Bending angle: 45°	50 s	^38^

The mass load sensitivity of the artificial muscle during actuation was tested. By recording CVs with different masses as a load, it was found that for both anodic and cathodic current peaks’ intensities decrease while the corresponding potential becomes more electropositive as the load is increased (Fig. 3a). The movement’s amplitude gets less visible as more load is added to the structure (Video S2). These changes in specific electrochemical parameters can be attributed to a decrease of the ionic migration. The actuation process is shown as a consequence of energy minimization taking place and therefore by adding a load this process is somehow disturbed, the ionic exchange between the conducting polymer and electrolyte being modified. Simultaneously, a limitation of the ability to actuate of the artificial muscle occurs as the load increases. As is the case for natural muscles, the device can undergo fatigue processes and after a certain weight value is reached it cannot make the difference between the loads. The sensitivity was 7.2 ± 0.33 mV/mg (for oxidation, Fig. 3b) and − 3.2 ± 0.23 mV/mg (for reduction, Fig. 3c). These results are in a good agreement with the theoretical model developed by Otero’s research team established for a material constituted by electrochemical reaction-driven molecular machines under galvanostatic conditions. The evolution of the consumed energy by the reaction, and the potential reached (as one of the components of the consumed energy) follow a linear dependence with the load mass, respecting the Eqs. (1) and (2)^39,40^:

1 $$E={C}_{1}+{C}_{2}m$$

2 $$U(t)={C}_{3}(t)+{C}_{4}(t)m$$

The experimental potential reached by the artificial muscle at any reaction time can be obtained from the chronopotentiograms previously shown (Fig. 4a,c) while the experimental energies consumed by the system during the electrochemical reactions can be calculated from the product of the potential reached and the charge consumed at any reaction time^41^. Figure 4a,c show, respectively, the potential reached by the artificial muscle after application of a square current wave of ± 0.5 mA for 3 s correlated with the consumed energy during the electrochemical reactions for each load. Both sensing values fit the theoretical model, obtaining the mechanical sensor calibration lines. The slopes (6.3 ± 0.7 and − 4.8 ± 0.72 mV/mg from Fig. 4b,d; 3.2 ± 0.27 and 2.5 ± 0.11 μJ/mg from Fig. 5a,b) represent the sensitivity of the sensor. From these lines, the weight supported by the fibrillary artificial muscle can be measured. In both cases, a difference in the oxidation and reduction slopes is founded, leading to an energetic asymmetry. This effect has been justified considering the different types of macroscopic volume changes of the polymer (swelling-relaxation consumes a different amount of energy than shrinking-compaction) and was expected from the actuation results by observing the extension of the curling movement. When there are no attached loads, the oxidation process tends to be more energetically favored (Fig. 4c), leading to deep curling movements (Fig. 2a). The asymmetry is also due to the exchange of solvent molecules to balance the osmotic pressure^42^. Similar characterization procedure tackle in this work can be found in other reported works such as Martinez and Otero^43^, however the actuator configurations are very different. In this case, the new material consists in freestanding electrospun microribbons (flat fibers with about 1 μm in width each) covered with a thin layer of gold (⁓ 200 nm) and PPy (⁓ 200 nm). So, here we have hundreds of bilayer actuators in one mesh strip (with sizes of about 0.1 × 2 cm width x length) than a classical bilayer actuator based on PPy coated flexible substrate. This makes that the up-dated actuator to presents increased mechanical sensitivity (fibers sensitivity is about 7 mV/mg for oxidation and − 4 mV/mg for reduction while the film sensitivity was 1 mV/mg for oxidation and 0.67 mV/mg for reduction^43^) Plus, this kind of artificial muscle is suitable for light weight actuators, even if it is soft and light (weighing less than 20 μg) it can uphold more than 21.1 mg.

The effect of the load modification on the ionic migration processes was followed by EIS. The weak migration of the electrolyte molecules (associated with minimization of diffusive processes and charge transfer resistance increase) at high load was demonstrated by estimating the electrode parameters for EIS measurements (displayed in Table 2). No significant changes in the charge transfer resistance and double layer capacitance of PPy/electrode interface (R_2_ and CPE_2_) under mechanical stress appear. In contrast, an important difference between the charge transfer resistance and double layer capacitance of PPy/electrolyte interface (R_1_ and CPE_1_) obtained with or without added mass can be identified (for R_1_ being almost 3 times higher for maximum load than unloaded muscle and almost 7 times higher for CPE_1_). Likewise, analyzing the n factor, it seems that the electrode crosses from a Warburg to a capacitor behavior by rising the load. This means that the incorporation of electrolyte ions into PPy film decreases by increasing the load. Moreover, a diffusion coefficient (D) for each weight was estimated considering a geometrical model (Eq. 3) suitable for a conducting polymer film with a finite thickness (L) and an ionic diffusion time constant (τ)^44^:

**Table 2:** Electrode parameters for each weight clung on the artificial muscle.

Element	0 mg	5.3 mg	10.6 mg	15.8 mg	21.1 mg
R_1_, Ω	60	67	101	133	164
CPE_1_, S	0.00081	0.00021	0.00015	0.00013	0.00012
n	0.57	0.74	0.77	0.77	0.77
τ, s	0.002	0.18	0.22	0.26	0.29
D (cm^2^/s, 10^–10^)	845	9.4	7.7	6.5	5.8

3 $$\tau =\frac{{L}^{2}}{D}$$

The large difference between the estimated diffusion coefficients of unloaded and loaded artificial muscle lead to the idea that an important part of the muscle mechanical sensitivity can be attributed to electrolyte ion migration, the process being reduced by increasing the load. As expected, this ionic inhibition causes also a decrease of movement amplitude.

It was demonstrated that the devices can simultaneously work as sensors and artificial muscles and a future work will aim to expand the mechanical sensitivity range (even more than 21.1 mg) of such materials, increasing their applicability.

## Conclusions

A fiber-based architecture of artificial muscles based on electrospun microribbons covered with polypyrrole was found to have intrinsic mechanical sensing capabilities, thus being close to both the morphology and functionality of natural animal muscles. The reversible electrochemical reactions of polypyrrole while immersed in a LiClO_4_ electrolyte under galvanostatic control lead to both actuation and sensing functionality. A reversible anti-clockwise/clockwise curling movement is observed for this particular structure—the actuation function. When different masses were employed as loads for the artificial muscle, mechanical sensing properties were found—the sensing function, while its the actuation performances are decreasing by increasing the weight. Thus, specific electrochemical parameters were depending on the load until a saturation occurs (in this particular case a mass of 21.1 mg), fitting the theoretical model previously developed. The mechanical sensitivity of the artificial muscles was found to be approximately 7 mV/mg for oxidation and -4 mV/mg for reduction (depending the registration method) in 0–21.1 mg weight range. These artificial muscle structures denote their potential as constitutive materials for a new generation of dual actuator-sensor soft electrochemical devices. This added function will allow complex machines to execute finely tuned tasks which need appropriate feedback from the sensorial system without the need of an external sensor monitor. This would update the former actuation-only artificial muscles towards new fibrillary self-sensing ones, coming closer to animal muscles.

## Methods

### Materials and equipments

For preparing the electrospun microribbon nets, nylon 6/6 (pellets, Sigma Aldrich, St. Louis, MO, USA; CAS number 32131-17-2) and formic acid (ACS reagent, ≥ 88.0%, Sigma Aldrich, St. Louis, MO, USA; CAS number 64-18-6) were used. Further, the conducting polymer was synthesized using lithium perchlorate (LiClO_4_, battery grade, dry, 99.99% metals basis, Aldrich, St. Louis, MO, USA; CAS number 7791-03-9), pyrrole (for synthesis, Sigma-Aldrich, St. Louis, MO, USA; CAS number 109-97-7), and acetonitrile (for HPLC, gradient grade, ≥ 99.9%, Honeywell, Charlotte, NC, USA; CAS number 75-05-8) as solvent. A gold target (99.99% purity, Kurt J. Lesker) and Ar (99.9999% purity, Linde) were employed for the metallization process. All solutions were prepared by using ultrapure water generated in a Milli-Q equipment (18.2 MΩ cm).

The procedure to cover the metalized microribbons with PPy, involved the use a Parstat 2273 Princeton Applied Research for electropolymerizing the monomer. The metalized microribbons attached on stainless steel frames were used as working electrode (WE), a platinum mesh as counter electrode (CE) and a commercial saturated calomel electrode as reference electrode (RE). For the electrochemical characterization, a MultiAutolab M101 potentiostat/galvanostat equipped with NOVA 2.1.4 software was used. The electrochemical cell was based on strips taken from the PPy coated microribbon net and used as WE, a platimum plate used as CE and a commercial Ag/AgCl electrode as RE. For the metallization process, a Torr DC sputtering equipment was employed.

### Preparation of PPy based fibrillary artificial muscle

The PPy based fibrillary artificial muscles were prepared according to a procedure described in a previous work^27^. At first, nylon 6/6 microribbons were prepared from a precursor solution by electrospinning in controlled environmental conditions and collecting on copper frames. In a second step, the electrospun ribbons were covered with a thin layer of gold by sputtering and later submerged into a monomeric solution, consisting in 0.2 M pyrrole and 0.1 M LiClO_4_ in acetonitrile and 2% water. Then, the metalized nets were used as working electrodes in the setup for potentiostatic electropolymerization of pyrrole, by applying a constant potential of 0.872 V for 120 s.

The preparation of such mesh strips is highly reproducible if the fiber density of the mesh and the strip’s width is preserved. The fiber density of such electrospun meshes can be kept the same by maintaining the same electrospinning parameters (especially fiber collecting time) and it can be checked by recording the transmission coefficient using a UV–Vis spectrophotometer. Further, two strips can be verified if they have the same electrochemical characteristics by recording control CVs and compare the results. In this work electroactive freestanding meshes with sizes of 2 × 3 cm length × width were prepared and, over time, samples were tested in this configuration, following the described procedure.

### Characterization

**Figure 7:**
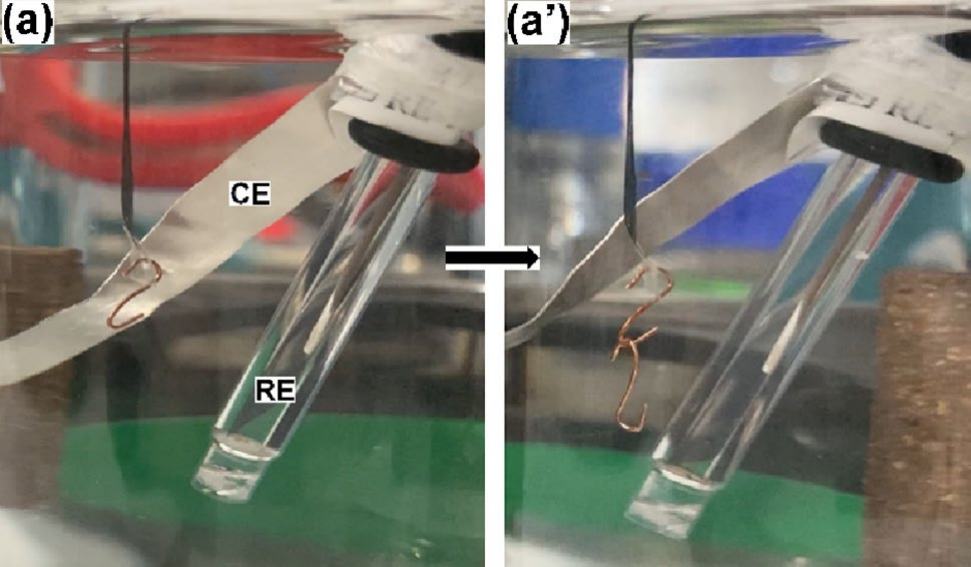
Experimental set-up used during characterization. An electrochemical cell containing an Ag/AgCl reference electrode (RE), a platinum plate counter electrode (CE) and the PPy coated microribbon strip as working electrode holding (**a**) 6 mg and (**b**) 12 mg.

After preparation, the PPy coated microribbon webs were cut in rectangular strips of 0.1 × 2 cm width x length and submerged in a 0.1 M LiClO_4_ aqueous solution. Then, using the microribbon strips as electrodes cyclic voltammetry (CV) experiments were performed using − 0.60 and + 0.60 V as potential limits and a scan rate of 100 mV s^−1^. Several cycles were performed allowing the system to remove a possible previous material memory and promoting the exchange of chloride from the electrolyte^45–47^. When a stationary response is obtained, the material’s electroactivity data is stored as a reference before the experimental series. The actuation performances of the artificial muscle were tested through cyclic voltammetry experiments considering the same parameters as those mentioned above. In a further step the sensing properties of the artificial muscle were investigated by recording consecutive CVs while sweeping the potential between − 0.60 and + 0.60 V at a scan rate of 100 mV s^−1^, while various weights were clung to the fibrillary strip (as can be seen in Fig. 7). Likewise, an anodic and a cathodic chronoamperometric response were obtained when the artificial muscle was subjected to successive square current waves by applying a constant current of ± 0.5 mA for 3 s each. The square current pulses were repeatedly applied while attaching the masses to the microribbon strip. Corresponding results were acquired by recording the electrochemical impedance spectroscopy (EIS) plots, EIS being a suitable technique for describing the ionic diffusion processes, when different loads were hung on the artificial muscle. The ionic diffusion behavior was investigated by applying a polarization potential of − 0.20 V, in the 0.1–100,000 Hz frequency range, at 10 points per decade and an AC voltage amplitude of 10 mV. The experimental data were interpreted by using a ZView 2 (Scribner Assoc.) fitting program, considering an electrical equivalent circuit (Fig. 6c) which was also employed to estimate the electrode parameters. The weights used during the experiment were prepared by cutting a copper wire and bending it in a S-shape in order to be attached to the artificial muscle. Since every cut wire had a weight of 6 mg, up to four masses were consecutively hung leading to masses of 6, 12, 18 and 24 mg respectively. In the sensitivity estimations, the buoyancy of the weight was taken into consideration by calculating the relative mass of the load of about 5.3, 10.6, 15.8 and 21.1 mg. The first mass was attached to the microribbons by using a piece of adhesive tape, Fig. 7 showing the experimental design used during experiments.

## Supplementary Information

Supplementary Legends.

Supplementary Video 1.

Supplementary Video 2.

## Data Availability

The datasets generated and analyzed during the current study are available from the corresponding author on reasonable request.
