# Supplementary material for: Bioinspired polypyrrole based fibrillary artificial muscle with actuation and intrinsic sensing capabilities
Source: Sci Rep. 2022 Sep 2;12:15019. doi: 10.1038/s41598-022-18955-6 (PMC9440232; doi:10.1038/s41598-022-18955-6)
Supplement: Supplementary file 1 — Supplementary Legends. [file 41598_2022_18955_MOESM1_ESM.docx]

**Supplementary Information**

Video S1 – Artificial muscle actuation.

Video S2 – Artificial muscle holding a piece of 6 mg.
